# Viruses and Evolution – Viruses First? A Personal Perspective

**DOI:** 10.3389/fmicb.2019.00523

**Published:** 2019-03-19

**Authors:** Karin Moelling, Felix Broecker

**Affiliations:** ^1^Institute of Medical Microbiology, University of Zurich, Zurich, Switzerland; ^2^Max Planck Institute for Molecular Genetics, Berlin, Germany; ^3^Department of Microbiology, Icahn School of Medicine at Mount Sinai, New York, NY, United States

**Keywords:** evolution, RNA world, ribozymes, origin of life, viruses

## Abstract

The discovery of exoplanets within putative habitable zones revolutionized astrobiology in recent years. It stimulated interest in the question about the origin of life and its evolution. Here, we discuss what the roles of viruses might have been at the beginning of life and during evolution. Viruses are the most abundant biological entities on Earth. They are present everywhere, in our surrounding, the oceans, the soil and in every living being. Retroviruses contributed to about half of our genomic sequences and to the evolution of the mammalian placenta. Contemporary viruses reflect evolution ranging from the RNA world to the DNA-protein world. How far back can we trace their contribution? Earliest replicating and evolving entities are the ribozymes or viroids fulfilling several criteria of life. RNA can perform many aspects of life and influences our gene expression until today. The simplest structures with non-protein-coding information may represent models of life built on structural, not genetic information. Viruses today are obligatory parasites depending on host cells. Examples of how an independent lifestyle might have been lost include mitochondria, chloroplasts, *Rickettsia* and others, which used to be autonomous bacteria and became intracellular parasites or endosymbionts, thereby losing most of their genes. Even *in vitro* the loss of genes can be recapitulated all the way from coding to non-coding RNA. Furthermore, the giant viruses may indicate that there is no sharp border between living and non-living entities but an evolutionary continuum. Here, it is discussed how viruses can lose and gain genes, and that they are essential drivers of evolution. This discussion may stimulate the thinking about viruses as early possible forms of life. Apart from our view “viruses first”, there are others such as “proteins first” and “metabolism first.”

## Ribozymes and Viroids

The origin of life on Earth has recently regained attention following the discovery of exoplanets within possible habitable zones (Kasting et al., 1993). The astronomical number of expected exoplanets suggests that there is a high statistical chance that life has also evolved somewhere else. This possibility stimulates the thinking of how life started on the early Earth, which may help to extrapolate to other planets.

Life presumably started simple. This is plausible, but is an assumption. The smallest known bacteria are still rather large. One of the smallest known metabolically autonomous bacterial species is *Pelagibacter ubique* with about 1,400 genes (Giovannoni et al., 2005). Genome reduction of *Mycoplasma mycoides* by systematic deletion of individual genes resulted in a synthetic minimal genome of 473 genes (Hutchison et al., 2016). Can one consider simpler living entities?

There are elements with zero genes that fulfill many criteria for early life: ribozymes, catalytic RNAs closely related to viroids. They were recovered *in vitro* from 10^15^ molecules (aptamers), 220 nucleotides in length, by 10 rounds of selection. Among the many RNA species present in this collection of quasispecies RNAs were catalytically active members, enzymatically active ribozymes. The sequence space for 220-mer RNAs is about 3 × 10^132^ (Eigen, 1971; Wilson and Szostak, 1999; Brackett and Dieckmann, 2006).

The selected ribozymes were able to replicate, cleave, join, and form peptide bonds. They can polymerize progeny chemically, allow for mutations to occur and can evolve. One molecule serves as catalyst, the other one as substrate. Replication of ribozymes was demonstrated in the test tube (Lincoln and Joyce, 2009). Ribozymes can form peptide bonds between amino acids (Zhang and Cech, 1997). Thus, small peptides were available by ribozyme activity. Consequently, an RNA modification has been proposed as peptide nucleic acid (PNA), with more stable peptide bonds instead of phosphodiester bonds (Zhang and Cech, 1997; Joyce, 2002). Replication of RNA molecules can be performed chemically from RNA without polymerase enzymes. In addition, deoxyribozymes can form from ribonucleotides (Wilson and Szostak, 1999). Thus, DNA can arise from RNA chemically, without the key protein enzyme, the reverse transcriptase.

An entire living world is possible from non-coding RNA (ncRNA) before evolution of the genetic code and protein enzymes. Ribozymes naturally consist of circular single-stranded RNAs (Orgel, 2004). They lack the genetic triplet code and do not encode proteins. Instead, they exhibit structural information by hairpin-loops that form hydrogen bonds between incomplete double strands, and loops free to interact with other molecules. They represent a quasispecies in which many species of RNA may form, such as ribozymes, tRNA-like molecules, and other ncRNAs. RNAs within such a pool can bind amino acids. Ninety different amino acids have been identified on the Murchison meteorite found in Australia, while on Earth only about 20 of them are used for protein synthesis (Meierhenrich, 2008). Where formation of ribozymes occurred on the early Earth is a matter of speculation. The hydrothermal vents such as black smokers in the deep ocean are possibilities where life may have started (Martin et al., 2008). There, temperature gradients and clay containing minerals such as magnesium or manganese are available. Pores or niches offer possibilities for concentration of building blocks, which is required for chemical reactions to occur. Interestingly, also ice is a candidate for ribozyme formation and chemical reactions. Ice crystals displace the biomolecules into the liquid phase, which leads to concentration, creating a quasicellular compartmentalization where *de novo* synthesis of nucleotide precursors is promoted. There, RNA and ribozymes can emerge, which are capable of self-replication (Attwater et al., 2010).

tRNA-amino acid complexes can find RNAs as “mRNAs.” Such interactions could have contributed to the evolution of the genetic code. This sequence of events can lead to primitive ribosome precursors. Ribozymes are the essential catalytic elements in ribosomes: “The ribosome is a ribozyme” (Cech, 2000), supplemented with about a hundred scaffold proteins later during evolution. The proteins have structural functions and contribute indirectly to enzymatic activity. Are these ribosome-bound ribozymes fossils from the early Earth? Small peptides can be formed by ribozymes before ribosomes evolved, whereby single or dimeric amino acids may originate from the universe (Meierhenrich, 2008).

Small peptides with basic amino acids can increase the catalytic activity of ribozymes as shown *in vitro* (Müller et al., 1994). Such proteins are known as RNA-binding proteins from RNA viruses that protect the RNA genome, with motifs such as RAPRKKG of the nucleocapsid NCp7 of HIV (Schmalzbauer et al., 1996). Peptides can enhance the catalytic activity of ribozymes up to a 100-fold (Müller et al., 1994). Such peptides of RNA viruses serve as chaperones that remove higher ordered RNA structures, allowing for more efficient interaction of RNA molecules and increasing transcription rates of RNA polymerases (Müller et al., 1994). Ribonucleoproteins may have also been functionally important during the evolution of ribosomes (Harish and Caetano-Anolles, 2012).

**FIGURE 1:**
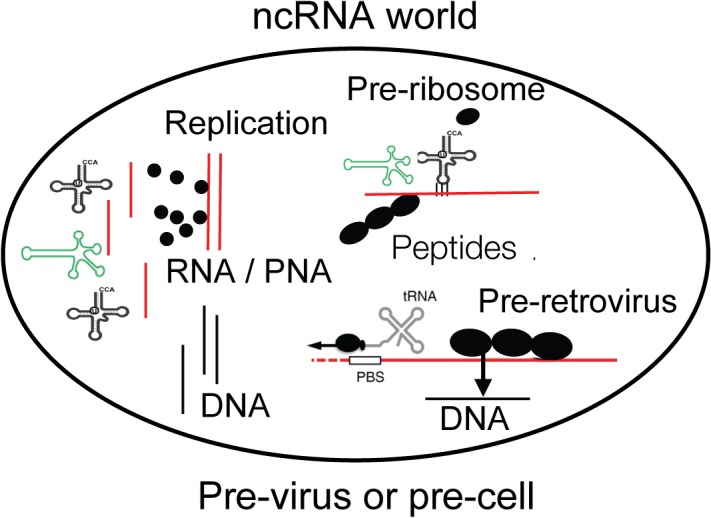
A compartment is shown with essential components of life as discussed in the text. Non-coding RNA (ncRNA), ribozymes or viroids, can perform many steps for life without protein-coding genes but only by structural information. Individual amino acids are indicated as black dots and may be available on Earth from the universe. DNA may have existed before retroviruses. The compartment can be interpreted as pre-virus or pre-cell. Viroid, green; RNA, red; DNA, black.

These pre-ribosomal structures are also similar to precursor-like structures of retroviruses. Reverse transcription can be performed by ribozymes chemically. This action does not necessarily require a protein polymerase such as the reverse transcriptase. Similarly, deoxyribonucleotides can arise by removal of an oxygen without the need of a protein enzyme (a reductase) as today, and allow for DNA polymerization (Wilson and Szostak, 1999; Joyce, 2002). The same elements of the precursors for ribosomes are also building blocks of retroviruses, which may have a similar evolutionary origin (Moelling, 2012, 2013). tRNAs serve as primers for the reverse transcriptase, and the sequence of promoters of transposable elements are derived from tRNAs (Lander et al., 2001). The ribozymes developed into more complex self-cleaving group II introns with insertion of genes encoding a reverse transcriptase and additional proteins (Moelling and Broecker, 2015; Moelling et al., 2017) (Figure 1).

It came as a surprise that the genomes of almost all species are rich in ncDNA, transcribed into ncRNAs but not encoding proteins, as evidenced, for instance, by the “Encyclopedia of DNA Elements” (ENCODE) project. ncDNA amounts to more than 98% of the human DNA genome (Deveson et al., 2017). Higher organisms tend to have more non-coding information, which allows for more complex modes of gene regulation. The ncRNAs are regulators of the protein-coding sequences. Highly complex organisms such as humans typically have a high number of ncRNA and regulatory mechanisms. ncRNA can range from close to zero in the smallest bacteria such as *Pelagibacter ubique* to about 98% in the human genome.

RNA viruses such as the retrovirus HIV harbor ncRNAs for gene regulation such as the *trans*-activating response element (TAR), the binding site for the Tat protein for early viral gene expression. Tat has a highly basic domain comprising mostly Lys and Arg residues, resembling other RNA binding proteins. ncRNA also serves on viral RNA genomes as ribosomal entry sites, primer binding sites or packaging signals. DNA synthesis depends on RNA synthesis as initial event, with RNA primers as starters for DNA replication, inside of cells as well as during retroviral replication, proving a requirement of RNA (Flint, 2015).

The number of mammalian protein-coding genes is about 20,000. Surprisingly, this is only a fifth of the number of genes of bread wheat (Appels et al., 2018). Tulips, maize and other plants also have larger genomes, indicating that the number of genes does not necessarily reflect the complexity of an organism. What makes these plant genomes so large, is still an open question. Could the giant genomes possibly be the result to breeding of plants by farmers or gardeners?

According to Szostak there are molecules which appear like relics from the RNA world such as acetyl-CoA or vitamin B12, both of which are bound to a ribonucleotide for no obvious reason – was it “forgotten” to be removed? (Roberts and Szostak, 1997; Szostak et al., 2001; Szostak, 2011). Perhaps the connected RNA serves as structural stabilizer. Lipid vesicles could have formed the first compartments and enclosed ribozymes, tRNAs with selected amino acids, and RNA which became mRNA. Is this a pre-cell or pre-virus (Figure 1)?

Patel et al. (2015) demonstrated that the building blocks of life, ribonucleotides, lipids and amino acids, can be formed from C, H, O, P, N, S in a “one pot” synthesis. This study can be regarded as a follow-up study of the classical Urey-Miller *in vitro* synthesis of biomolecules (Miller, 1953; Miller and Urey, 1959). Transition from the RNA to the DNA world was promoted by the formation of the reverse transcriptase. The enzyme was first described in retroviruses but it is almost ubiquitous and found in numerous cellular species, many of which with unknown functions (Simon and Zimmerly, 2008; Lescot et al., 2016). It is an important link between the RNA and the DNA worlds. The name reverse transcriptase is historical and irritating because it is the “real” transcriptase during the transition from the RNA to the DNA world. Similarly, the ribonuclease H (RNase H) is an essential enzyme of retroviruses (Mölling et al., 1971). The RNase H turned out to be one of the five most frequent and ancient proteins (Ma et al., 2008) that belongs to a superfamily of more than sixty different unique representatives and 152 families with numerous functions (Majorek et al., 2014).

Some of the many tRNAs can become loaded with amino acids. There are viruses containing tRNA-like structures (TLS), resembling these early RNAs (Dreher, 2009). The TLS of these viruses typically bind to a single amino acid. TLS-viruses include plant viruses, such as Turnip yellow mosaic virus, in Peanut clump virus, Tobacco mosaic virus (TMV), and Brome mosaic virus. Only half a tRNA is found in Narnaviruses of fungi. The amino acids known to be components of tRNA-like viruses are valine, histidine and tyrosine. The structures were also designated as “mimicry,” enhancing translation (Dreher, 2009, 2010). They look like “frozen” precursor-like elements for protein synthesis. This combination of a partial tRNA linked to one amino acid can be interpreted as an evolutionary early step toward protein synthesis, trapped in a viral element.

**FIGURE 2:**
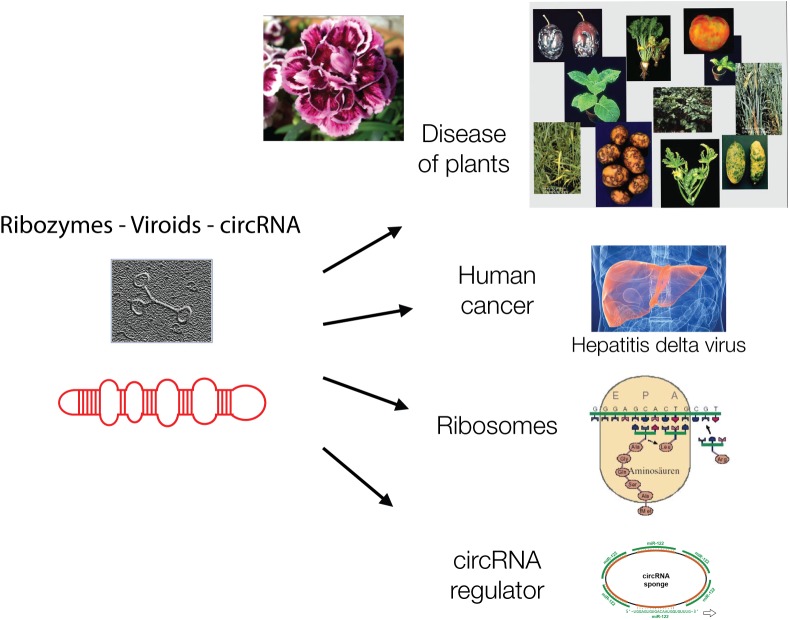
Viroids are hairpin-loop structures and are shown schematically and as electron micrograph. Viroids are, like ribozymes, without genetic information and play major biological roles today in plant diseases, in carnation flowers, in liver cancer, as catalyst of protein synthesis in ribosomes and as circular regulatory RNAs, as “sponges” for other regulatory RNAs.

Ribozymes are related to the protein-free viroids. Viroids are virus-like elements that belong to the virosphere, the world of viruses (Chela-Flores, 1994). Viroids lack protein coats and therefore were initially not designated as viruses but virus-like viroids when they were discovered in 1971 by Theodor Diener. He described viroids as “living fossils” (Diener, 2016) (Figure 2).

From infected potatoes, Diener isolated the Potato spindle tuber viroid (PSTVd) whose genome was about a 100-fold smaller than those of viruses known at that time. The viroids known today are ranging from 246 to 467 nucleotides. They contain circular single-stranded RNA, are protein-free and self-replicating with no genetic information, but only structural information in the form of hairpin-loops (Riesner et al., 1979). They can generate copies of themselves in the appropriate environment. They were designated as the “frontiers of life” (Flores et al., 2014).

The knowledge of virus composition was based on TMV and its crystallization by Wendell Stanley in 1935 (Pennazio and Roggero, 2000). The genome of TMV is protein-coding single-stranded RNA of about 6,400 nucleotides that is enclosed by a rod-like protein coat. Viroids, in contrast, do not encode proteins and lack coats but they are closely related to viruses. Viroids can lose their autonomy and rely on host RNA polymerases to replicate, are capable of infecting plants and many are economically important pathogens. There are two families, the nucleus-replicating *Pospiviroidae* such as PSTVd and the chloroplast-replicating *Avsunviroidae* like the Avocado sunblotch viroid (ASBVd). Their replication requires host enzymes. Thus, autonomy is replaced by dependence on host enzymes and an intracellular lifestyle.

Most viroids are often enzymatically active ribozymes – yet they are examples that this trait can get lost as a result of changing environmental conditions. Loss of ribozyme activity is a functional, not a genetic loss. Only the nuclear variants, the *Pospiviroidae*, can lose their ribozyme activity and use the cellular RNase III enzyme for their replication. In contrast, the *Avsunviroidae* are still active hammerhead ribozymes. Thus, inside the nucleus of a host cell, the enzymatic RNA function can become unnecessary. Not genes, but a function, the catalytic activity, gets lost.

Viroids did apparently not gain genes but cooperated for a more complex lifestyle. For example, Carnation small viroid-like RNA (CarSV RNA) cooperates with a retrovirus and is accompanied by a homologous DNA generated by a reverse transcriptase. This enzyme presumably originates from a pararetrovirus of plants. Pararetroviruses package virus particles at a different stage during replication than retroviruses, the DNA, not the RNA. This unique combination between two viral elements has so far only been detected with CarSV in carnation flowers (Flores et al., 2005, 2014). Why did such a cooperation evolve – perhaps by breeding gardeners? RNA is sensitive to degradation; therefore, genetic increase and growth of the genome may not be favorable energetically – at least not in plants. Gain of function is, in this case, cooperation.

The circular RNA (circRNA) is related to ribozymes/viroids as a chief regulator of other regulatory RNAs, a “sponge” absorbing small RNAs. Micro RNAs (miRNAs) are post-transcriptional regulators that are affected by the presence of circRNAs. circRNAs were detected in human and mouse brains and testes as well as in plants. They can bind 70 conserved miRNAs in a cell and amount up to 25,000 molecules (Hansen et al., 2013). Their structure is reminiscent of catalytically active ribozymes.

There is an exceptional viroid that gained coding information and entered the human liver (Taylor, 2009). The viroid is known as hepatitis delta virus (HDV). It has the smallest genome of any known animal virus of about 1,680 nucleotides. It has properties typical of viroids, since it contains circRNA, forms similar hairpin-loops and replicates in the nucleus using host enzymes. Two polymerases have to redirect their specificity from DNA to RNA to generate the HDV genome and antigenome. Both of them have ribozyme activity. In contrast to other ribozymes, HDV encodes a protein, the hepatitis delta antigen (HDVAg) that occurs in two forms, the small-HDVAg (24 kDa) supporting replication and the large-HDVAg (27 kDa) that helps virion assembly. The gene was presumably picked up from the host cell by recombination of HDV’s mRNA intermediate with a host mRNA. Transmission depends on a helper virus, the Hepatitis B virus (HBV), which delivers the coat (Taylor, 2009) Does packaging by a helper virus protect the genome and thereby allow for a larger viroid to exist?

In plants, viroids may not be able to become bigger possibly due to their sensitivity to degradation – but they cannot become much smaller either. Only a single viroid is known that is completely composed of protein-coding RNA with triplets (AbouHaidar et al., 2014). Viroids and related replicating RNAs are error-prone replicating units and the error frequency imposes a certain minimal size onto them, as they would otherwise become extinct. This mechanism has been described as “error catastrophe,” which prevents survival (Eigen, 1971, 2013). The viroids and related RNAs are the smallest known replicons. Smaller ones would become extinct in the absence of repair systems.

In summary, RNA can catalyze many reactions. Protein enzymes which may have evolved later have higher catalytic activities. Ribozymes are carriers of information, but do not require coding genes. Information is stored in their sequence and structure. Thus, replication of an initial RNA is followed by flow of information, from DNA to RNA to protein, as described the Central Dogma (Crick, 1968). Even an information flow from protein to DNA has been described for some archaeal proteins (Béguin et al., 2015). The DNA-protein world contains numerous ncRNAs with key functions. ncRNA may serve as a model compound for the origin of life on other planets. Hereby not the chemical composition of this molecule is of prime relevance, but its simplicity and multifunctionality. Furthermore, RNA is software and hardware in a single molecule, which makes it unique in our world. There are other scenarios besides the here discussed “virus-first,” such as “protein-first”, “metabolism-fist” or the “lipid world” (Segré et al., 2001; Andras and Andras, 2005; Vasas et al., 2010; Moelling, 2012). Some of these alternative concepts were built on phylogenomics, the reconstruction of the tree of life by genome sequencing (Delsuc et al., 2005). Surprisingly, it was Sir Francis Crick, one of the discoverers of the DNA double-helix, who stated that he would not be surprised about a world completely built of RNA. A similar prediction was made by Walter Gilbert (Crick, 1968; Gilbert, 1986). What a vision! Our world was almost 50 years later defined as “RNA-protein” world (Altman, 2013). One can speculate our world was built of ribozymes or viroids, which means “viruses first.”

## Spiegelman’s Monster

ncRNAs appear as relics from the past RNA world, before DNA, the genetic code and proteins evolved. However, ncRNA is essential in our biological DNA world today. It is possible to produce such ncRNA today in the test tube by loss of genic information from protein-coding RNA. This reduction to ncRNA was demonstrated *in vitro* with phage RNA. Phage Qβ genomic RNA, 4,217 nucleotides in length, was incubated in the presence of Qβ replicase, free nucleotides and salts, a rich milieu in the test tube. The RNA was allowed to replicate by means of the Qβ replicase. Serial transfer of aliquots to fresh medium led to ever faster replication rates and reduction of genomic size, down to 218 nucleotides of ncRNA in 74 generations. This study demonstrated that, depending on environmental conditions, an extreme gene reduction can take place. This experiment performed in 1965 was designated as “Spiegelman’s Monster.” Coding RNA became replicating ncRNA (Spiegelman et al., 1965; Kacian et al., 1972)!

Manfred Eigen extended this experiment and demonstrated further that a mixture containing no RNA to start with but only ribonucleotides and the Qβ replicase can under the right conditions in a test tube spontaneously generate self-replicating ncRNA. This evolved into a form similar to Spiegelman’s Monster. The presence of the replicase enzyme was still necessary in these studies. Furthermore, a change in enzyme concentration and addition of short RNAs or an RNA intercalator influenced the arising RNA population (Sumper and Luce, 1975; Eigen, 2013). Thus, the complexity of genomes depends on the environment: poor conditions lead to increased complexity and rich environments to reduced complexity.

The process demonstrated in this experiment with viral components indicates that reversion to simplicity, reduction in size, loss of genetic information and speed in replication can be major forces of life, even though this appears to be like a reversion of evolution. The experiment can perhaps be generalized from the test tube to a principle, that the most successful survivors on our planet are the viruses and microorganisms, which became the most abundant entities. Perhaps life can start from there again.

These studies raise the question of how RNA molecules can become longer, if the small polymers become smaller and smaller, replicate faster and outcompete longer ones. This may be overcome by heat flow across an open pore in submerged rocks, which concentrates replicating oligonucleotides from a constant feeding flow and selection for longer strands. This has been described for an increase from 100 to 1,000 nucleotides *in vitro*. RNA molecules shorter than 75 nucleotides will die out (Kreysing et al., 2015). Could a poor environment lead to an increase of complexity? This could be tested. Ribozymes were shown to grow in size by uptake of genes, as demonstrated for HDV (Taylor, 2009).

## Microbiome in the Human Intestine

An interesting recent unexpected example supporting the notion that environmental conditions influence genetic complexity, is the human gut microbiome. Its complexity increases with diverse food, while uniform rich food reduces its diversity and may lead to diseases such as obesity. Colonization of the human intestinal tract starts at birth. A few dozen bacterial and viral/phage species are conserved between individuals (core sequences) as a stable composition (Broecker et al., 2016c, 2017). Dysbiosis has been observed in several chronic diseases and in obesity, a loss of bacterial richness and diversity. Nutrition under affluent conditions with sugar-rich food contributes to obesity, which results in a significant reduction of the complexity of the microbiome. This reduction is difficult to revert (Cotillard et al., 2013; Le Chatelier et al., 2013). The gut microbiome in human patients with obesity is reminiscent of the gene reduction described in the Spiegelman’s Monster experiment: reduction of genes in a rich environment.

The reduction of the complexity of the microbiome is in part attributed to the action of phages, which under such conditions, defined as stress, lyse the bacteria. Fecal microbiota transplantation can even be replaced by soluble fractions containing phages or metabolites from the donor without bacteria (Ott et al., 2017). Analogously, the most highly complex microbiomes are found in indigenous human tribes in Africa, which live on a broad variety of different nutrients. It is a slow process, though, to increase gut microbiota complexity by diverse nutrition. The obesity-associated microbiota that survive are fitter and more difficult to counteract. Urbanization and westernization of the diet is associated with a loss of microbial biodiversity, loss of microbial organisms and genes (Segata, 2015).

To understand the mechanism and driving force for genome reduction, deletion rates were tested by insertion of an indicator gene into the *Salmonella enterica* genome. The loss of the indicator gene was monitored by serial passage in rich medium. After 1,000 generations about 25% of the deletions caused increased bacterial fitness. Deletions resulted in smaller genomes with reduced or absence of DNA repair genes (Koskiniemi et al., 2012). Gene loss conferred a higher fitness to the bacteria under these experimental conditions.

## Mimiviruses – Gain or Loss?

**FIGURE 3:**
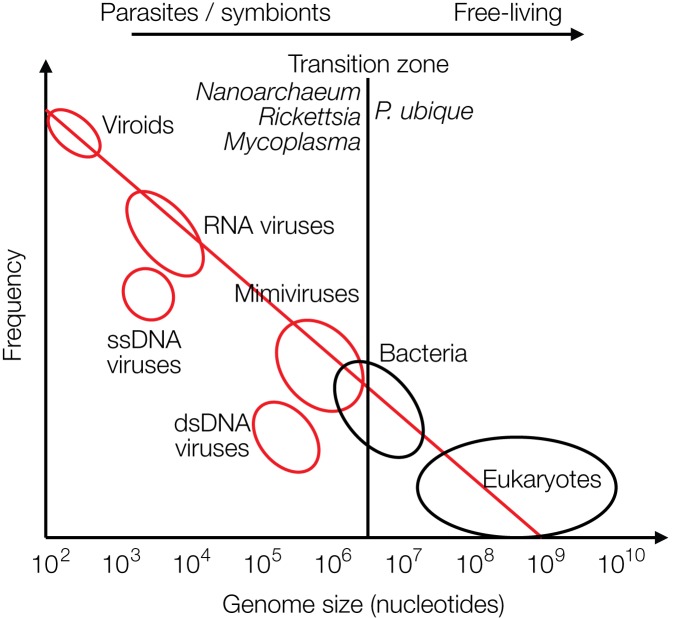
Size distribution of viruses (red circles), free-living bacteria and eukaryotes (black circles) are shown relative to their frequencies. The transition zone between parasites or symbionts versus free-living species is indicated by a black line. The transition is not a sharp borderline as shown by the circles and as discussed in the text (modified from Holmes, 2011).

The recently discovered mimiviruses and other giant viruses are worth considering for understanding the evolution of life with respect to the contribution of viruses. Their hosts are, for example, *Acanthamoeba*, *Chlorella*, and *Coccolithus* algae (*Emiliania huxleyi*), but also corals or sponges as discussed more recently. Mimiviruses were first discovered in cooling water towers in Bradford, United Kingdom in 2003 with about 1,000 genes, most of which unrelated to previously known genes. Mimiviruses have received attention because they contain elements that were considered hallmarks of living cells, not of viruses, such as elements required for protein synthesis, tRNAs and amino acid transferases. The mimiviruses harbor these building blocks as incomplete sets not sufficient for independent protein synthesis as bacteria or archaea can perform, preventing them from leading an autonomous life (La Scola et al., 2003, 2008). They are larger than some bacteria. Giant viruses can be looked at as being on an evolutionary path toward a cellular organism. Alternatively, they may have evolved from a cellular organism by loss of genetic information (Nasir and Caetano-Anolles, 2015). Giant viruses have frequently taken up genes from their hosts by horizontal gene transfer (HGT) (La Scola et al., 2008; Nasir and Caetano-Anolles, 2015; Colson et al., 2018). A graph on genome sizes shows that mimiviruses and bacteria overlap in size, indicating a continuous transition between viruses and bacteria and between living and non-living worlds (based on Holmes, 2011) (Figure 3). Other giant viruses, such as megaviruses, were discovered in the ocean of Chile with 1,120 genes. Most recently the Klosneuvirus was identified in the sewage of the monastery Klosterneuburg in Austria in 2017 with 1.57 million (mio) basepairs (Mitch, 2017). *Pithovirus sibericum* is the largest among giant viruses discovered to date with a diameter of 1.5 microns, a genome of 470,000 bp with 467 putative genes, 1.6 microns in length, and it is presumably 30,000 years old as it was recovered from permafrost in Siberia (Legendre et al., 2014). The smaller *Pandoraviruses* with 1 micron in length have five times larger genomes, 2,500,000 bp (Philippe et al., 2013) (Figure 3).

The giant viruses can even be hosts to smaller viruses, the virophages, reminiscent of bacteriophages, the viruses of bacteria. These virophages such as Sputnik are only 50 nm in size with 18,343 bp of circular dsDNA and 21 predicted protein-coding genes. They replicate in viral factories and consume the resources of the mimivirus, thereby destroying it. Some, virophages can even integrate into the genome of the cellular host and can be reactivated when the host is infected by giant viruses. Thus, giant viruses suggest that viruses are close to living entities or may have been alive (La Scola et al., 2008; Fischer and Hackl, 2016).

In biology it is common to distinguish between living and dead matter by the ability to synthesize proteins and replicate autonomously. The giant viruses may be considered as missing link between the two, because they harbor “almost” the protein synthesis apparatus. The transition from living to the non-living world is continuous, not separated by a sharp borderline (Figure 3).

Viruses are not considered alive by most of the scientific community and as written in textbooks, because they cannot replicate autonomously. Yet some of the giant viruses are equipped with almost all components of the protein synthesis machinery close to bacteria suggesting that they belong to the living matter (Schulz et al., 2017). The ribozymes may have been the earliest replicating entity. Perhaps also other viruses were initially more independent of the early Earth than they are today. As described in Figure 1 there may have been initially no major difference between an early virus or an early cell. Only later viruses may have given up their autonomous replication and became parasites – as has been described for some bacteria (see below).

Efforts have been made to identify the smallest living cell that is still autonomously replicating. Among the presumably smallest naturally occurring bacteria is *Pelagibacter ubique* of the SAR11 clade of bacteria (Giovannoni, 2017), which was discovered in 1990. It is an alpha-proteobacterium with 1,389 genes present ubiquitously in all oceans. It can reach up to 10^28^ free living cells in total and represents about 25% of microbial plankton cells. Very little of its DNA is non-coding. It harbors podophage-type phages, designated as “pelagiphage” (Zhao et al., 2013). This small bacterium was designated as the most common organism on the planet. Why is it so successful? This autonomous bacterium is smaller than some parasitic giant viruses. Craig Venter, who first succeeded in sequencing the human genome, tried to minimize the putative smallest genome of a living species, from *Mycoplasma mycoides*, a parasitic bacterium that lives in ruminants (Gibson et al., 2008, 2010). His group synthesized a genome of 531,000 bp with 473 genes, 149 of them (32%) with unknown functions (Hutchison et al., 2016). Among the smallest parasitic living organisms is *Nanoarchaeum equitans*. It is a thermophile archaeon which lives at 80°C and at pH 6 with 2% salt (Huber et al., 2003). Its genome has a size of 490,000 bp and encodes 540 genes. *N. equitans* is an obligate symbiont of a bigger archaeon, *Ignicoccus* riding on it as on a horse, hence the name (Huber et al., 2003).

The world of viruses covers a range of three logs in size of their genomes: from zero genes to about 2,500 genes amounting to about 2,500,000 bp of DNA. The zero-gene viroids are about 300 bases in length (Figure 3).

The virosphere is the most successful reservoir of biological entities on our planet in terms of numbers of particles, speed of replication, growth rates, and sequence space. There are about 10^33^ viruses on our planet and they are present in every single existing species (Suttle, 2005).

There is no living species without viruses! Viruses also occur freely in the oceans, in the soil, in clouds up to the stratosphere and higher, to at least 300 km in altitude. They populate the human intestine, birth canal, and the outside of the body as protective layer against microbial populations. Microbes contain phages that are activated during stress conditions such as lack of nutrients, change in temperatures, lack of space and other changes of environmental conditions.

## Retroviruses as Drivers of Evolution

One of the most earth-shaking papers of this century was the publication of the human genome sequence (Lander et al., 2001). About half, possibly even two-thirds of the sequence are composed of more or less complete endogenous retroviruses (ERVs) and related retroelements (REs) (de Koning et al., 2011). REs amplify via copy-and-paste mechanisms involving a reverse transcriptase step from an RNA intermediate into DNA. In addition, DNA transposable elements (TEs) move by a cut-and-paste mechanism. The origin of REs is being discussed as remnants of ancient retroviral germline infections that became evolutionarily fixed in the genome. About 450,000 human ERV (HERV) elements constitute about 8% of the human genome consisting of hallmark retroviral elements like the *gag, pol, env* genes and flanking long terminal repeats (LTR) that act as promoters (Lander et al., 2001). Howard Temin, one of the discoverers of the reverse transcriptase, in 1985 already described endogenous retrovirus-like elements, which he estimated to about 10% of the human and mouse genome sequence (Temin, 1985). The actual number is about 45% as estimated today (Lander et al., 2001). In some genes such as the *Protein Kinase Inhibitor B* (*PKIB*) gene we determined about 70% retrovirus-related sequences (Moelling and Broecker, 2015). Is there a limit? Could it have been 100%? Retroviruses are estimated to have entered the lineage of the mammalian genome 550 million years ago (MYA) (Hayward, 2017). Older ERV sequences may exist but are unrecognizable today due to the accumulation of mutations.

ERVs undergo mutations, deletions or homologous recombination events with large deletions and can become as short as solo LTR elements, which are a few hundred bp in length – the left-overs from full-length retroviral genomes of about 10,000 bp. The LTR promoters can deregulate neighboring genes. Homologous recombination events may be considered as gene loss or gene reduction events. It is the assumption that the ERVs, which were no longer needed for host cell defense, were no longer selected for by evolution and consequently deleted as unnecessary consumers of energy.

Eugene Koonin points out that infection and integration are unique events occurring at a fast pace, while loss and gene reduction may take much longer time frames (Wolf and Koonin, 2013).

A frequent gene reduction of eukaryotic genomes is the loss of the viral envelope protein encoded by the *env* gene. Without a coat, retroviruses can no longer leave the cell and infect other cells. They lose mobility and become obligatory intracellular elements. Helper viruses can supply envelope proteins in *trans* and mobilize the viruses. TEs or REs can be regarded as examples of coat-free intracellular virus relics – or could it have been the other way round, perhaps precursors of full-length retroviruses? These elements can be amplified intracellularly and modify the host genomes by integration with the potential danger of gene disruption and genetic changes. REs can lead to gene duplications and pseudogene development, with one copy for stable conservation of acquired functions and the other one for innovations (Cotton and Page, 2005). Such duplications constitute large amounts of mammalian genomes (Zhang, 2003). Retroviruses have an RNase H moiety duplication, one of which serves as a catalytically inactive linker between the RT polymerase and the enzymatically active RNase H (Xiong and Eickbush, 1990; Malik and Eickbush, 2001; Moelling and Broecker, 2015; Moelling et al., 2017). This gene duplication dates back to 500 mio years (Cotton and Page, 2005).

Gene duplications are a common cause of cancer, which often occurs only in the genome of the cancer cell itself, less affecting offsprings. Myc, Myb, ErbB2, Ras, and Raf are oncogenes amplified in diverse types of human cancers (Vogelstein and Kinzler, 2002). The ability of retroviruses to integrate makes them distinct from endosymbionts which stay separate. Yet the net result is very similar, acquisition of new genetic information, which is transmitted to the next generation, if the germline is infected and endogenization of the virus occurred.

Viral integration is not limited to eukaryotic cells but also a mechanism in prokaryotes for maintenance of the lysogenic state of phages inside bacteria.

Also, for other eukaryotic viruses such as HBV, the envelope surface antigen BHsAg can be deleted, which leads to an obligatory intracellular life style for the virus, which especially in the presence of HCV promotes cancer (Yang et al., 2016).

HIV has been shown to rapidly lose one of its auxiliary genes, *nef*, originally for negative factor. The gene was lost within a rather low number of passages of the virus grown under tissue culture conditions by selection for high virus titer producing cells. Deletion of *nef* resulted in a significant increase of the virus titer in culture – hence the name. The *nef* gene product was of no need inside tissue culture cells, rather it was inhibitory for replication. However, it is essential for pathogenicity in animals, and subsequently *nef* was reinterpreted as “necessary factor” (Flint, 2015).

Also, the human hosts of HIV can lose a significant terminal portion of a seven transmembrane receptor in lymphocytes, the primary target cell for HIV entry and for virus uptake. This molecule, the CCR5 cytokine receptor is truncated by 32 carboxy-terminal amino acids (CCR5-Δ32), disabling the receptor functionally. The allele frequency of the mutant CCR5-Δ32 mutant is about 10% in the European population, making these people resistant to HIV infections (Solloch et al., 2017). This gene loss in Europeans has been shown to make the individuals resistant not only against HIV infection but also against malaria. This may have been the selective pressure in the past before HIV/AIDS arose. No side effect for humans lacking this gene has been described (Galvani and Slatkin, 2003).

Viruses have been proven to be drivers of evolution (Villarreal and Witzany, 2010), including the human genome, which by at least 45% is composed of sequences related to retroviruses. In addition, endogenized retroviruses supplied the syncytin genes that are essential for the development of the mammalian placenta, and allowed the growth of embryos without its rejection by the maternal immune system (Dupressoir et al., 2012). Thus, the same property which causes immunodeficiency in HIV-infected patients and leads to AIDS causes syncytia formation, cell fusion after infection by a retrovirus. Viruses have also been proposed to be at the origin of the evolution of adaptive immunity (Villarreal, 2009). Thus, viruses shaped genomes by supplying essential genes and mechanisms.

## Endogenization of Viruses

Endogenization of retroviruses has occurred in the mammalian genomes for at least 550 mio years (Hayward, 2017). If the integrated ERVs did not provide any selective advantage, they deteriorated and accumulated mutations with loss of function. This was directly proven by reconstruction of an infectious retrovirus from the consensus sequence of 9 defective endogenous virus sequences, designated as Phoenix. The virus was expressed from a constructed synthetic DNA clone in cell culture and formed virus particles identified by high resolution microscopic analysis (Dewannieux and Heidmann, 2013).

The koalas in Australia are currently undergoing endogenization of a retrovirus (koala retrovirus, KoRV) in “real time” and demonstrate possible consequences for immunity. In the early 1900s, some individuals were transferred to islands, including Kangaroo Island, close to the Australian mainland for repopulation purposes, as koalas were threatened to become extinct. Today, the majority of the koala population is infected by KoRV, which is closely related to the Gibbon ape leukemia virus (GALV). Yet, koalas isolated on Kangaroo Island are KoRV negative, which allows dating the introduction of KoRV into the koala population to about one hundred years ago. Many of the infected koalas fell ill and died, yet some populations became resistant within about 100 years, corresponding to about 10 generations. The koalas likely developed resistance due to the integrated DNA proviruses. The retrovirus is transmitted as exogenous as well as endogenous virus, similar to the Jaagsiekte sheep retrovirus (JSRV), whereby the endogenized viruses protect with a viral gene product, such as Env, against *de novo* infections by “superinfection exclusion” (Tarlinton, 2012). The contribution of retroviruses to the antiviral defense is striking, since all retroviral genes have analogous genes in the siRNA/RNAi defense mechanism of eukaryotic cells (Moelling et al., 2006).

## Viruses Protect Against Viruses

**FIGURE 4:**
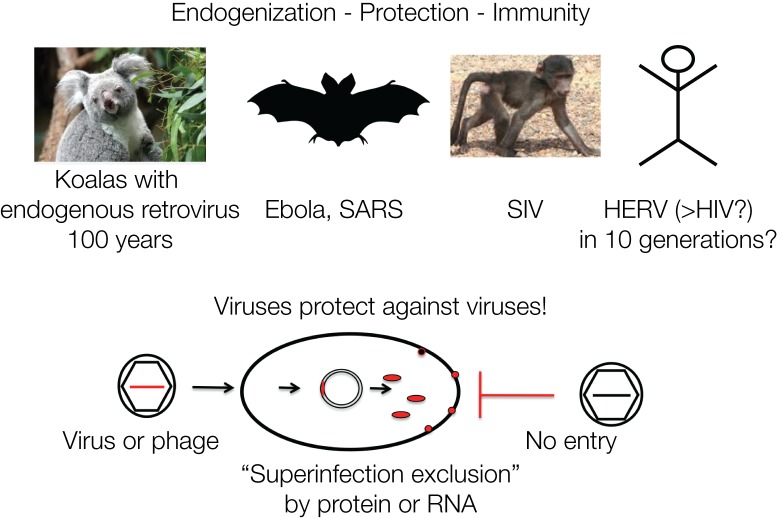
Viruses protect against viruses: retroviruses protect a cell against a new infection by a similar virus designated as “superinfection exclusion” or viral interference. This is mediated by viral gene products such as proteins or nucleic acids. Similarly, phages protect against phages: superinfection of bacteria is prevented by CRISPR/Cas RNA originating from previous infections. The mechanisms of defense against viruses and phages are analogous. Protection by viruses or phages against superinfections represents cellular defense and acquired immunity. The four examples are discussed in the text.

Retroviruses can protect against infection by other related viruses, for example, by expressing Env proteins that block cell-surface receptors (Villarreal, 2011). A comparable mechanism protects bacterial cells against DNA phages, by integrated phage DNA fragments that are transcribed into mRNA and hybridize to incoming new DNA phages and thereby lead to their destruction by hybrid-specific nucleases, CRISPR/Cas immunity (Charpentier and Doudna, 2013). It is often not realized that immunity acquisition in bacteria and mammalian cells follow analogous mechanisms (Figure 4).

Integration of retroviruses normally occurs in somatic cells after infection as an obligatory step during the viral life cycle. Infection of germline cells can lead to transmission to the next generation and ultimately result in inherited resistance. Endogenized retroviruses likely caused resistance to the exogenous counterparts. Similarly, resistance to Simian Immune Deficiency virus (SIV) in some monkey species may be explained by endogenization (Li et al., 2017, 2018). In the case of phages and their prokaryotic hosts the mechanism is described as CRISPR/Cas, which follow analogous principles of “endogenization” of incoming genetic material for subsequent exclusion.

One may speculate that HIV may also eventually become endogenized into the human genome. There is some evidence that HIV can infect human germline cells and can be transmitted to the embryonic genome (Wang et al., 2011). How long this may take is not known – 10 generations?

The loss of function of ERVs can occur by mutations, deletions of the *env* or other genes and ultimately all coding genes by homologous recombination, leaving behind only one LTR. The number of retrovirus-like elements add up to about 450,000, corresponding to 8% of the human genome (Lander et al., 2001; Cordaux and Batzer, 2009). The promoter regions were analyzed for their contribution to cancer by activating neighboring genes – as a consequence of a former retrovirus infection. Indeed, activated cellular genes by “downstream promotion” were identified in animal studies with activation of the *myc* gene as one of many examples, leading to chronic, not acute development of cancer (Ott et al., 2013). As a general mechanism for human cancer today the LTRs are, however, not identified as a major culprit. Most of the ERVs we find today have been integrated during evolution in introns or other regions where their presence is relatively harmless. Did the other ones result in death of the carriers which disappeared? The effects of LTRs on the expression levels of neighboring host genes was studied with the endogenous human virus, HERV-K, as a possible cause of cancer, but this appears not to be a general phenomenon (Broecker et al., 2016b). As shown for the koalas, ERVs can confer immunity to viral infections (Feschotte and Gilbert, 2012).

A related ERV, HERV-H, was shown to produce an RNA that keeps early embryonic cells pluripotent and even revert adult cells to regain pluripotency (Grow et al., 2015). Thus, the role of ERVs may be more complex than we presently know.

Transposable elements and REs that lost the ability of cellular transmission by deletion of the coat protein majorly contribute to genetic complexity of host cells. They are “locked” inside the cells and are major drivers of the increase of genetic complexity (Cordaux and Batzer, 2009). One could speculate that these intracellular elements are replication-incompetent retroviruses lacking coats (Lander et al., 2001). Bats transmit viruses such as Ebola and SARS coronavirus without suffering from disease (Beltz, 2018). Even RNA viruses such as Bornaviruses have been shown to integrate by illegitimate reverse transcription, possibly also supplying immunity against superinfection (Katzourakis and Gifford, 2010).

## Endosymbiosis

There are two prominent events that significantly contributed to the success of life and the formation of cells. Both of them are associated with gene reduction. This phenomenon may play a role for the evolution of viruses from autonomous to parasitic lifestyles. In the 1960s Lynn Margulis proposed an extracellular origin for mitochondria (Margulis, 1970, 1993). An ancestral cell, perhaps an archaeon, was infected by an anaerobic bacterium, which gave rise to mitochondria. Similarly, cyanobacteria formed the chloroplasts in modern plant cells. Mitochondria arose around 1.45 billion years ago (BYA) (Embley and Martin, 2006). Mitochondria and chloroplasts are the most striking examples for a change in lifestyle from autonomous bacteria to endosymbionts. This transition is often considered as extremely rare and a hallmark of evolution of life on our planet. However, there are many other obligate intracellular parasites such as *Rickettsia*, *Chlamydia trachomatis, Coxiella burnetii* (the causative agent of Q fever), *Mycobacterium leprae*, *M. tuberculosis*, and *M. mycoides* (Beare et al., 2006).

The change of lifestyle of the endosymbionts in the two cases of mitochondria and chloroplasts is striking. Both of them drastically reduced their genetic make-up. Mitochondria contain less than 37 genes, left from the original about 3,000 genes. Is endogenization of retroviruses, the ERVs, which are integrated into germline cells, related to endosymbiosis? Are these endosymbionts models for the transition from autonomous lifestyle to a parasitic life- which may have taken place with viruses?

A more recent typical example for a reductive evolution are *Rickettsia.* These bacteria were assumed for some time to be viruses because of their obligatory intracellular parasitic existence. *Rickettsia* have evolved from autonomously replicating bacteria. Reductive evolution of endosymbionts can yield bacteria with tiny genomes on the expense of autonomous extracellular life. Their genomes are 1.11 mio bp in length with about 834 protein-coding genes, and loss of 24% by reductive evolution (Ogata et al., 2001). *Rickettsia* may have some relationship with cyanobacteria, which are considered as the major symbionts.

Can one speculate that viruses may have been autonomous entities initially? Viroids may have undergone transition from autonomy to parasites, just as shown for mitochondria, chloroplasts or *Rickettsia*? To which extent have viruses been autonomous and independent of cellular metabolisms originally – and contributed to the origin of cells? Could they only later have lost their autonomy and become parasitic?

## Viruses and Oncogenes

Viruses are minimalistic in their composition and must have undergone stringent gene reductions (Flint, 2015). How small can their genomes become? Most coding RNA viruses still contain regulatory elements, ncRNA at the 3′ and 5′ terminal regions for ribosomal entry, protein synthesis, transcriptional regulation, and others.

A subgroup of retroviruses is an interesting example in respect to simultaneous loss and gain of genetic information. The oncogenic retroviruses or tumorviruses can recombine with cellular genes which under the promoters of retroviruses can become oncogenes and drivers of cancer. About a hundred oncogenes have been selected for in the laboratories and studied over decades for understanding the molecular mechanisms of cancer. Selection for growth advantages of the host cells led to the discovery of the fastest growth-promoting oncogenes we know today, such as Ras, Raf, ErbB or Myc, which are in part successful targets for anticancer drugs (Moelling et al., 1984).

These oncogenes were in most cases taken up by the retroviruses at the expense of structural (*gag*), replicating (*pol*) or envelope (*env*) genes, and are often expressed as fusion proteins with Gag. Thus, oncogenic retroviruses are obligatory intracellular defective viruses and were selected for in the laboratory by researchers for the oncogenes with the most potent growth promoting ability. They need the supply of replicatory genes in *trans* from co-infecting helper viruses to infect other cells (Flint, 2015). Retroviruses are able to pick up cellular genes, transfer and integrate them into neighboring cells. Some strains of Rous sarcoma virus maintain replication competent when carrying the cell-derived *src* (for sarcoma) oncogene encoding a protein of 536 amino acids that apparently can fit into the retroviral particle along with the full-size viral genome (Broecker et al., 2016a). Spatial reasons may have influenced the formation of oncogenic retroviruses and limited their size and thereby led to their defective phenotypes.

There are indications that the uncontrolled activity of (retro)transposons in germline cells can result in diseases such as male infertility – presumably by “error catastrophe,” caused by too many transposition events. In mammals, piRNAs tame transposon activity by means of the RNase H activity of PIWI proteins during spermatogenesis (Girard et al., 2006).

Only a minority of viruses are pathogens; most of them do not cause diseases. On the contrary, they are most important as drivers of evolution, as transmitters of genetic material, as innovative agents. In particular, the RNA viruses are the most innovative ones. Some of them are pathogenic and dangerous, such as HIV or influenza virus, or viroids in plants. RNA viruses are able to change so rapidly that the host immune system is unable to counteract the infection. Pathogenicity arises when environmental conditions change, for instance, when a virus enters a new organism or species.

Increase of cellular complexity by viruses is an important feature of evolution. Such major evolutionary changes are recently taken as arguments against the evolutionary theory by Charles Darwin who considered gradual changes, small increments by mutations as the main basis for selection and evolution. New criticism is addressing this thinking, considering larger changes as evolutionary drivers. Such changes arise by many complex phenomena such as endosymbiosis, infection by prokaryotes, viruses and fungi, recombination of genes, HGT, infections, sex. Dramatic changes such as endosymbiosis or pathogen infections extend Darwin’s concept of evolution.

## Conclusion

There are numerous examples for the contribution of viruses to the evolution of life since at least as long as 550 MYA (Hayward, 2017). But genetic noise through random mutations does not allow us to go back to the origin of life. It may not be impossible that the earliest compartment was indistinguishable, either a pre-cell or a pre-virus. By analogy one may speculate that at some point autonomous viruses gave up independence for an obligatory intracellular life – as has been described for mitochondria and chloroplasts but also intracellular bacteria such as *Rickettsia*. This speculation is based on the concept that early life must have started simple and with high genetic variability and then became more complex. But complexity can be given up for a less energy consuming lifestyle with small genomes and high speed of replication (Moelling, 2012, 2013). Therefore, the question may be repeated: “Are viruses our oldest ancestors?” Some fossil life can be partially reproduced *in vitro* by Spiegelman’s Monster and Eigen’s follow-up experiments, explaining the great surviving potential of simple ncRNA.

Viruses can be pathogens, but their recognition as primarily causing diseases is wrong. This notion is based on the history of viruses in medicine, as explained in a book entitled “Viruses: More Friends Than Foes” (Moelling, 2017). The scenario described here focuses on viruses as drivers of evolution.

The early RNA world gained interest 20–30 years ago as evidenced by the references provided above. Surprisingly, there are scientists who still believe in the “pansperm hypothesis” and think that retroviruses are of extraterrestric origin (Steele et al., 2018). The recent interest in the origin of life arose from the newly discovered exoplanets whose number increases daily – and which may be as numerous as 10^25^. Thus, pure statistics make some people believe that there is extraterrestrial life.

The extraterrestric life is mimicked in laboratories on Earth with many assumptions – perhaps this overview stimulates some thinking. The discussion presented here should be taken as concept about simple replicating and evolving entities possibly arising from different building blocks in other environments, with structure being more relevant than sequence.

## Author Contributions

Both authors wrote the manuscript and approved the final version.

## Conflict of Interest Statement

The authors declare that the research was conducted in the absence of any commercial or financial relationships that could be construed as a potential conflict of interest.
